# Supplementary material for: Non-contact detection of pyrethroids widely used in vector control by Anopheles mosquitoes
Source: PLoS One. 2024 Jul 12;19(7):e0298512. doi: 10.1371/journal.pone.0298512 (PMC11244766; doi:10.1371/journal.pone.0298512)
Supplement: S1 File — (DOCX) [file pone.0298512.s001.docx]

**Non-contact detection of pyrethroids widely used in vector control by *Anopheles* mosquitoes**

KAMBOU *et al*.

**Supplementary materials**

**Overview of the protocol**

These experiments tested whether an adult female mosquito would respond when presented with a continuous flow of the bottle headspace containing either an insecticide or a control (i.e., acetone) stimulus. A positive response was recorded when a resting mosquito took off within 30s of exposure to the airflow.

The insecticide stimulus involved filtered air being passed through a glass bottle in which a known quantity of insecticide had been dissolved in acetone and allowed to evaporate. The control stimulus involved air passed through a bottle where the same quantity of acetone than in the test bottle was placed and allowed to evaporate.

The temperature of the airflow to which mosquitoes were exposed was controlled to be at either 25°C or 35°C.

Experiments recorded the responses of *Anopheles gambiae* mosquitoes from the two strains, kis and kdrkis, insecticide sensitive and resistant, respectively.

On any one day, a single strain of mosquito was used and a single dose of one insecticide was used to prepare the insecticide stimulus treatment.

Four groups of 10 adult female mosquitoes of a similar age (~ 4-8 days post-emergence) were prepared each day. The four groups were used to test the two-by-two combination of stimulus and temperature treatments.

|  |  | Stimulus | |
| --- | --- | --- | --- |
|  |  | Control | Insecticide |
| Temperature (°C) | 25 | n = 10 | n = 10 |
|  | 35 | n = 10 | n = 10 |

The 10 females in each group were individually exposed to the same combination of stimulus and temperature treatments and their response recorded. For logistical reasons it was easier to test the stimulus treatments at one temperature and then at the other temperature. To avoid bias due to sequence of treatments tested, the initial temperature treatment to be tested was varied between days, as was the initial stimulus treatment (insecticide or control) within temperature treatments.

The experimental design has a split-plot structure where individual days are whole-plots determined by the strain and dose of treatments, with sub-plots being the stimulus and temperature treatments within each day.

The split-plot design outlined above was replicated at different times for three different insecticides; alpha-cypermethrin, deltamethrin and permethrin. In total, the responses of 3822 female mosquitoes were recorded from 384 groups of 10 mosquitoes over 96 different days. For 364 of the groups, the responses of all 10 females were recorded. In 19 cases the responses for nine of the 10 females were known due to handling or data recording errors. There was also one group where a response was recorded for 11 females.

**Spatial and temporal variation of experiments**

Although the exact same two Kisumu strains of *Anopheles gambiae*, kis and kdrkis, were used throughout the experiments, there was temporal and spatial differences in the experiments. The different insecticide treatments were tested in three blocks of experiments varying over time and space. The first block of experiments tested responses to permethrin and was performed in Montpellier (France). The second and third blocks were performed in Bobo-Dioulasso (Burkina Faso) and tested responses to deltamethrin and alpha-cypermethrin, respectively. Hence, in order to avoid confounding effects of the response variable arising from these temporal and spatial differences, we have opted to perform the statistical analyses for each block of insecticides independently.

**Proportion of females responding to the control stimulus varied over blocks of experiments**

The proportion of female mosquitoes responding to the control stimulus varied in the three blocks of experiments associated with the three insecticides (S1 Fig.).

The overall proportion of females responding to the control stimulus in the experiments involving alpha-cypermethrin and deltamethrin were similar, with both being greater than for those involving permethrin; 0.325 (32.5%), 0.296 (29.6%) and 0.126 (12.6%), respectively (S1 Fig.a,b).

**
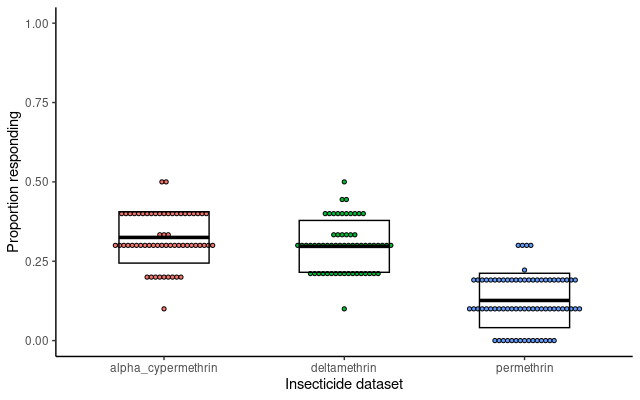
 a**

**b**

**
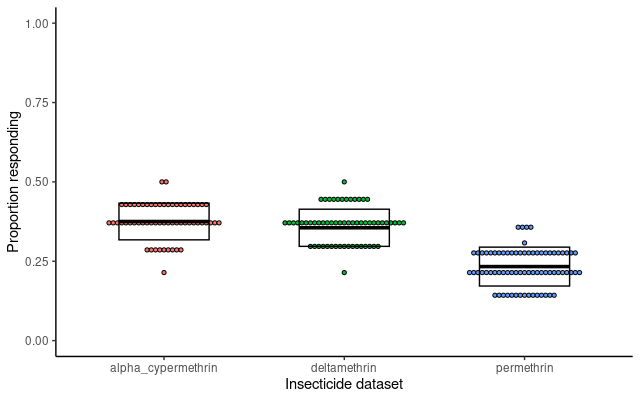
**

S1 Fig. Proportion of female mosquitoes responding to negative control stimulus grouped by insecticide treatment. Each dot represents the proportion of females responding in each group of 10 mosquitoes exposed to the control stimulus. Boxes represent the mean (± 1 standard deviation). (a) observed data (b) the observed data following a '+2/+2' transformation.

There was no a priori reason to expect these differences as in each case, females were exposed to air flow arising from a glass bottle rinsed with acetone and allowed to evaporate. However, the experiments involving alpha-cypermethrin and deltamethrin were conducted in Burkina Faso, while those with permethrin were conducted in France. Furthermore, this spatial variation was confounded with temporal variation due to the different times and generations of mosquito used. Consequently, we analysed each block of experiments separately where responses to the insecticide stimulus were compared to those of the matching control treatment within the same block of experiments.

*Proportions transformed*

Our aim was to test whether female mosquitoes were more likely to respond to the headspace of insecticide treated bottles than to a matching headspace of a bottle without the insecticide. To do so, we compared the proportion of females responding in an insecticide treatment, pI, with the proportion responding in the matching control treatment, pC. The ratio of these proportions estimates the 'relative risk' (RR) of responding to the insecticide stimulus;

RR = pI / pC

Values of RR > 1 (hence, log(RR) > 0, as in S2 Fig indicate mosquitoes are more likely to respond when exposed to the insecticide stimulus.

In the block of experiments involving permethrin there were several groups of control mosquitoes where none of the 10 females responded to the control stimulus (S1 Fig.a). In such cases the proportion responding equals zero, pC = 0, and the value of relative of risk is infinite, RR = ∞, and cannot be used in analyses. This situation was avoided by transforming the data.

Here we applied the '+2/+2' transformation proposed by Agresti & Coull (1). In this case a value of two is added to both: the number of successes and the number of failures in each group of mosquitoes; thus, the total number of trials increases by four. This transformation causes proportions to 'shrink' away from the lower and upper bounds and towards the mid-point. Hence in a group where no mosquitoes responded to the control stimulus, pC = (0/10) = 0, transforms to pC = (2/14) = 0.142, allowing a finite value of relative risk to be calculated. S1 Fig.b shows the transformed values of the observed data in Figure S1a. The transformed data are used in all subsequent analyses.

**Proportion of females responding to the control stimulus varied little within blocks of experiments**

While the proportion of females responding in the control treatments varied among the three blocks of experiments, these proportions varied little within blocks.

For each block of experiments, the proportion of females responding in the control stimulus treatments varied little across the combination of different dose, temperature and strain treatments (S2 Fig.a-c). Note 'dose' on the x-axis of the Fig refers to the dose of insecticide in the matching insecticide stimulus treatment; in each control treatment, mosquitoes were exposed to the headspace from a glass bottle rinsed with acetone and allowed to evaporate. As we were primarily interested in the pattern of responses to the dose of insecticide used, rather than the specific response to any particular dose of insecticide, the effect of dose was treated as a continuous variable when analyzing the data.

**a**
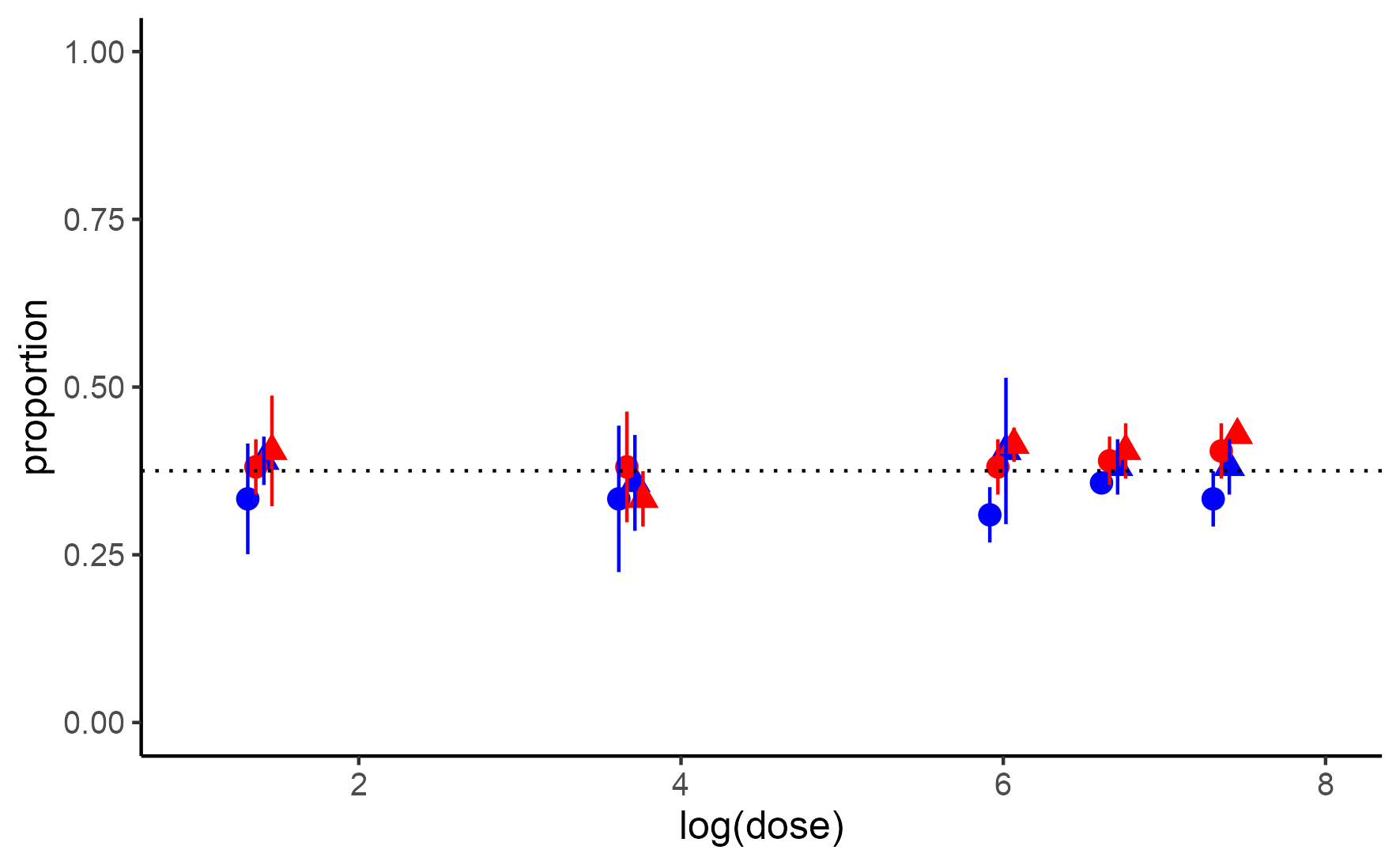
 **Alpha cypermethrin**

**b Deltamethrin**

**
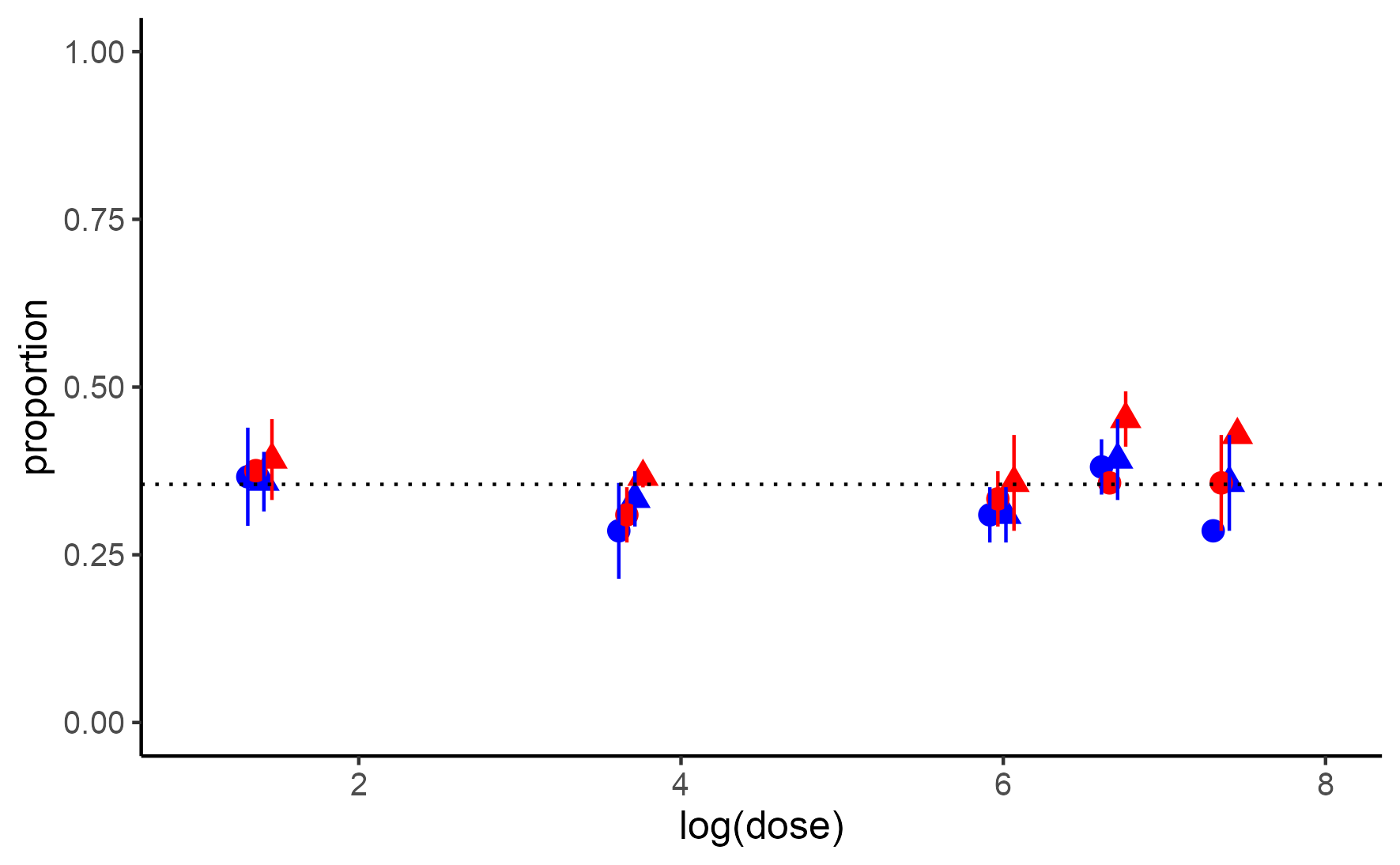
**

**c**
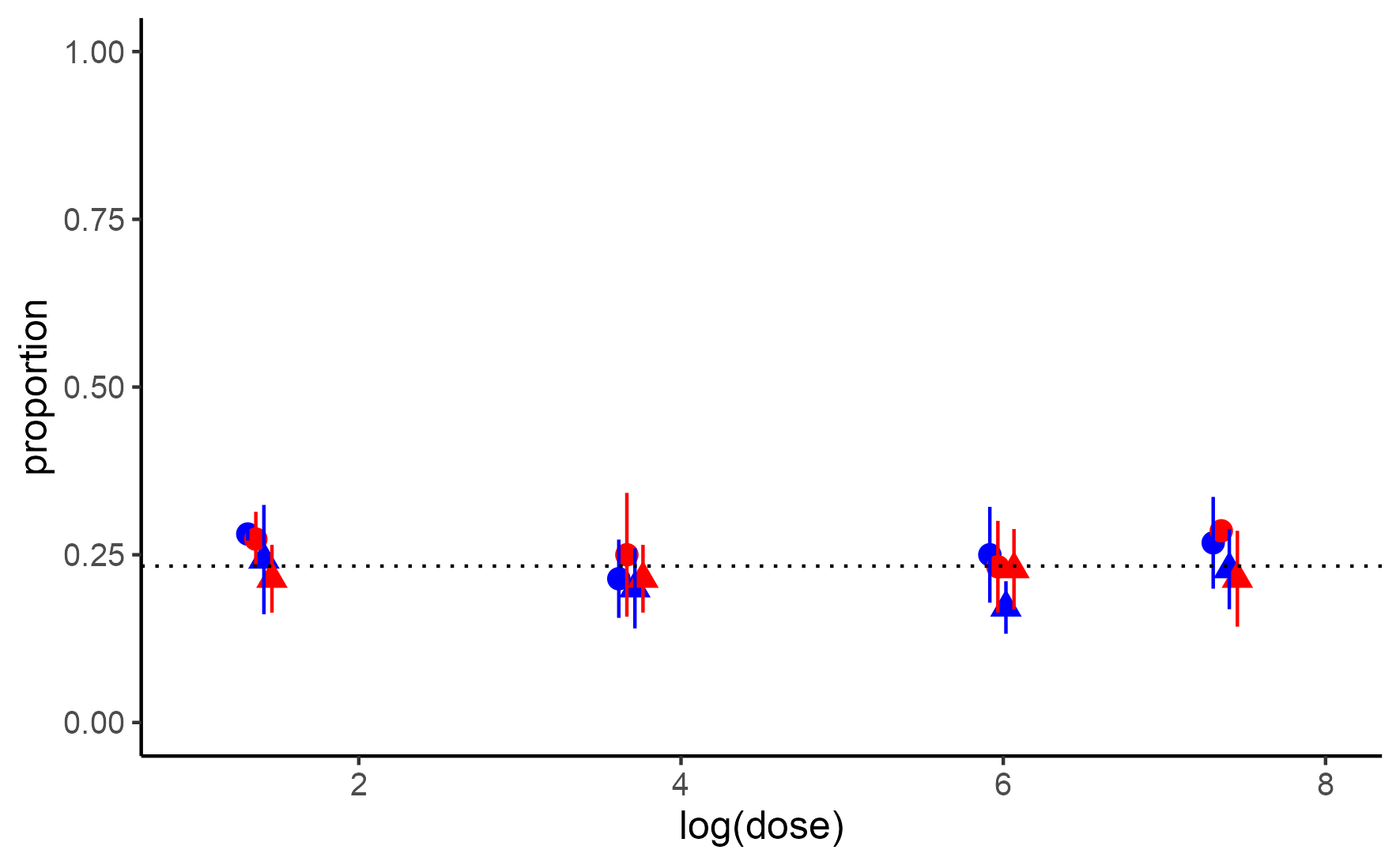
 **Permethrin**

**
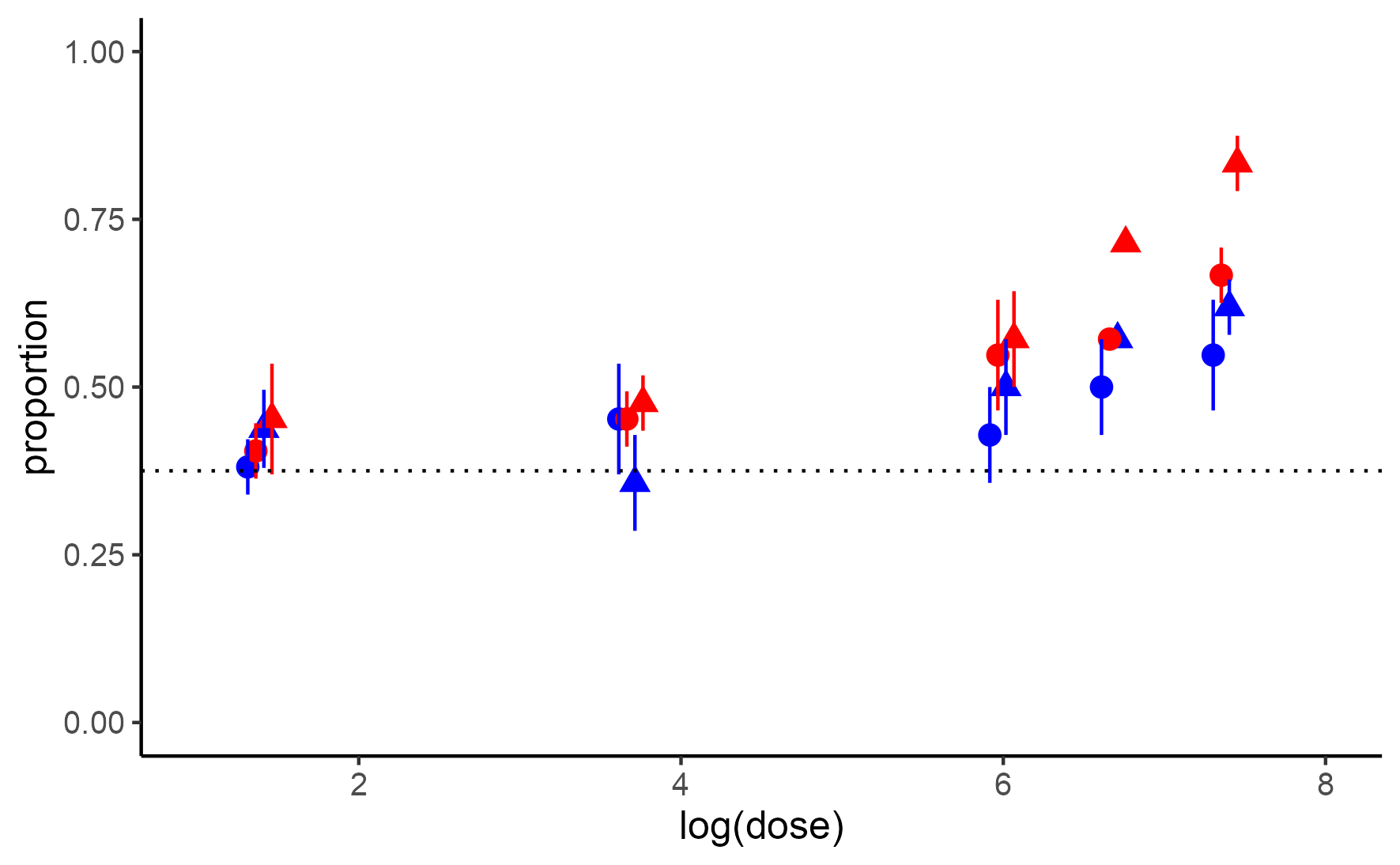
 d Alpha cypermethrin**

**e Deltamethrin**


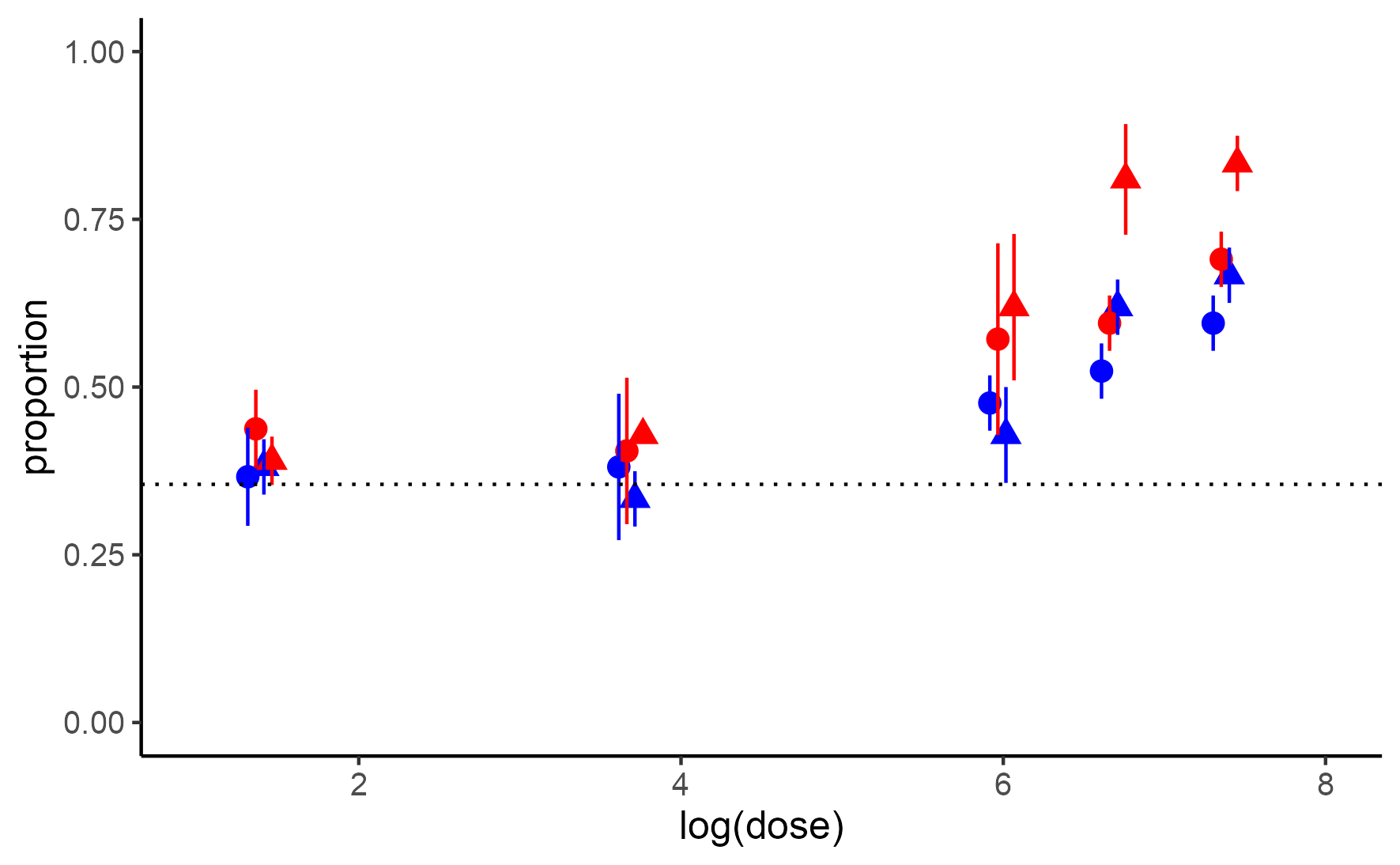


**f**
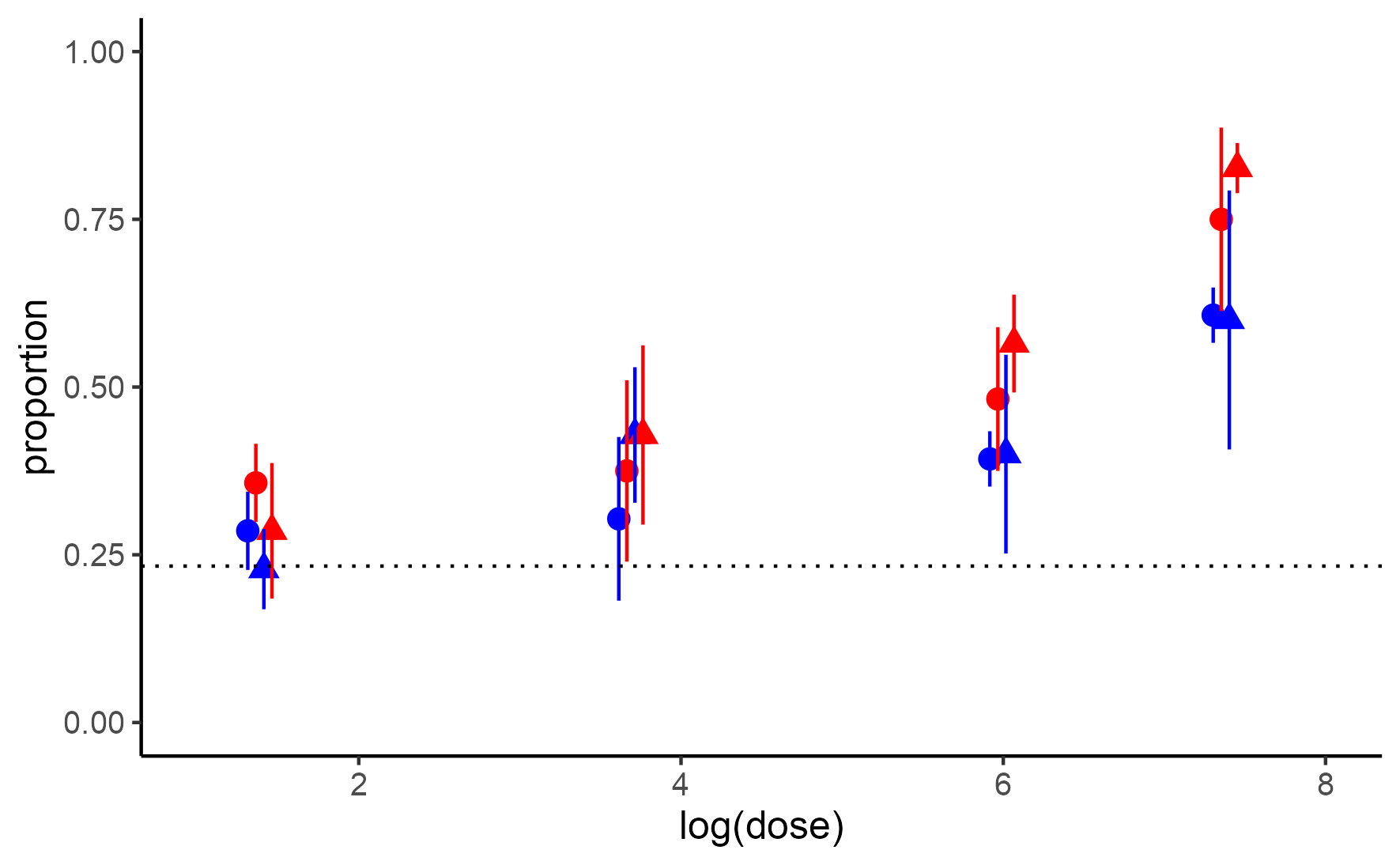
 **Permethrin**

S2 Fig. Proportions of female mosquitoes responding in each dataset grouped by insecticide (columns) and plotted against the log(dose) of insecticide used in the insecticide stimulus treatment. Panels (a-c) shows the proportion of females responding in the control treatments, (d-f) shows the proportion responding in the insecticide stimulus treatments, Data for the 25°C treatment are in blue, and red for the 35°C treatment. Circles are for the strain kdrkis and diamonds are for the strain kis. The dotted horizontal lines in (a-f) are the estimated proportion of females responding in the control treatments.

Generalized linear models allowing for random effects ('glmer') were used to analyse the binomial proportion of mosquitoes responding in the control treatments, taking the split-plot nature of the experimental design into account. These models generally reported warnings of singularity in the model fit and estimated zero variance for the random effects, which could be due to the limited variation in the data being analysed.

Re-running the models without the random effects, i.e., as 'glm' models, yielded same log-likelihood, but without the 'cost' of a degree of freedom taken to estimate the variance of the random effects. Consequently the 'glm' models without random effects were retained for subsequent analyses.

The treatment effects of strain, temperature and dose were then analysed for their effects on the binomial proportion of females responding in the control treatments. This was done with a series of 'glm' models ranging from a fully factorial three-way Anova with the effects strain, temperature and dose, down to the 'null model' where only the intercept is estimated. The results of these models were compared by AIC. This allowed identification of the 'best model' with the lowest AIC and 'near models' that can be considered as describing the data equally well as the 'best model' by virtue of an AIC within a difference of two from that of the 'best model' (2)

The 'best model' describing the binomial proportion of females responding in the control treatments in the datasets for deltamethrin and alpha-cypermethrin was the 'null model' in which only the intercept was estimated. The 'null model' was also in the 'near models' for the permethrin dataset. Consequently the 'null model' was adopted for describing the proportion of females responding in the control treatments for each dataset; these were estimated as 0.375, 0.355 and 0.233 for the alpha-cypermethrin, deltamethrin and permethrin, respectively (S2 Fig.a-c).

*Offset models*

As outlined above, the ratio of binomial proportions responding in matching insecticide stimulus (pI) and control stimulus (pC) treatments estimates the 'relative risk' (RR) of responding to the insecticide stimulus;

RR = pI / pC.

This could be calculated for each group of females in an insecticide stimulus treatment and that of the matching control treatment. However, the analyses above indicate the best estimates for the control data were from the values of the 'intercept', i.e., the overall proportion of mosquitoes responding in the control treatments of each insecticide dataset. Adopting the latter values has the advantages of (i) reducing the overall variance in estimates of relative risk, and, (ii) making the variance to be analysed due only to variation in the response of females to the insecticide stimulus treatment.

Taking the logarithm of relative risk allows the contribution of the insecticide and control stimulus treatments to be separated,

log (RR) = log(pI / pC) = log(pI) - log(pC)

Such that the data can be re-arranged and expressed in the following form;

log(pI) = model + log(pC)

where the model is to be defined and log(pC) is called an offset term. In the following analyses we used linear regression models to analyse log(pI).

**Proportion of females responding to the insecticide stimulus treatments**

In contrast to the control treatments and for each dataset, the proportion of females responding in the insecticide stimulus treatments varied across treatments, in particular increasing as the dose of insecticide in the stimulus treatment increased (S2 Fig.d-f).

A series of regression models with an offset term analysed log(pI) with models ranging from a fully-factorial three-way model with the effects strain, temperature and dose, down to the 'null model' only estimating the 'intercept'.

As for the control data, these models were compared by AIC to identify the 'best model' and 'near models' having an AIC within a value of two of the 'best model'.

The 'best model' identified for each of the three insecticide datasets was different. However, there was one model found within the 'nearly models' of the three datasets. This model was chosen as the 'consensus' model providing the best overall description of the whole data;

log(pI) = strain + temperature + log(dose) + log(pC)

For the three datasets there was a significant effect of dose (p < 0.001), such that the proportion of females responding to the insecticide stimulus treatment increased relative to that in the control stimulus treatments as the quantity of insecticide used to prepare the stimulus increased.

There was also a significant effect of temperature in each dataset (p < 0.005), such that, the relative proportion of females responding in the insecticide stimulus treatment was greater when the airflow was maintained at 35°C, rather than at 25°C.

Finally, although the term 'strain' was retained for the 'consensus model' it was only significant (p < 0.05) in the alpha-cypermethrin dataset and where the relative proportion of females responding in the insecticide stimulus treatment was greater for the kis strain than for the strain kdrkis.

**Field *Anopheles gambiae sl* susceptible and resistant genotypes: *ace1*, *kdr* east and *kdr* west**

S1 Table. *ace1* genotypes distribution in *Anopheles gambiae sl*

|  | Genotype *rr* | Genotype *rs* | Genotype *ss* |
| --- | --- | --- | --- |
| *An. arabiensis* | 1 | 20 | 52 |
| *An. gambiae* | 0 | 14 | 112 |
| *An. coluzzii* | 0 | 12 | 46 |

S2 Table. *kdr* East genotypes distribution in *Anopheles gambiae sl*

|  | Genotype *rr* | Genotype *rs* | Genotype *ss* |
| --- | --- | --- | --- |
| *An.arabiensis* | 1 | 41 | 35 |
| *An.gambiae* | 0 | 18 | 45 |
| *An.coluzzii* | 0 | 22 | 106 |

S3 Table. *kdr* West genotypes distribution in *Anopheles gambiae sl*

|  | Genotype *rr* | Genotype *rs* | Genotype *ss* |
| --- | --- | --- | --- |
| *An.arabiensis* | 10 | 31 | 36 |
| *An.gambiae* | 47 | 10 | 6 |
| *An.coluzzii* | 30 | 35 | 66 |

NB: 15 mosquitoes were excluded because species was not identified. An additional 18 mosquitoes were excluded, as their resistance status was not determined for *kd*r and/or *ace1*

S4 Table. Combinations of *kdr* insecticide resistance genotypes at both loci: *kdr*-East and *kdr*-West. [rr] = homozygous resistant; [rs] = heterozygous sensitive; [ss] = homozygous sensitive; [NA] = missing data.

| Genotypes | RR | RS | SS | NA |
| --- | --- | --- | --- | --- |
| East [NA] — West[*rr*] | 3 | 0 | 0 | 0 |
| East [NA] — West [*rs*] | 0 | 0 | 0 | 1 |
| East [*rr*] — West [NA] | 1 | 0 | 0 | 0 |
| East [*rr*] — West [*ss*] | 1 | 0 | 0 | 0 |
| East [*rs*] — West [rr] | 5 | 0 | 0 | 0 |
| East [rs] — West [rs] | 0 | 2 | 0 | 0 |
| East [rs] — West [ss] | 0 | 19 | 0 | 0 |
| East [ss] — West [NA] | 0 | 0 | 0 | 2 |
| East [ss] — West [rr] | 53 | 0 | 0 | 0 |
| East [ss] — West [rs] | 0 | 17 | 0 | 0 |
| East [ss] — West [ss] | 0 | 0 | 87 | 0 |

References

1. Agresti A, Coull BA. Approximate Is Better than ‘Exact’ for Interval Estimation of Binomial Proportions. Am Stat. 1998;52(2):119–26.

2. Burnham KP, Anderson DR, editors. Model Selection and Multimodel Inference [Internet]. New York, NY: Springer New York; 2004. Available from: http://link.springer.com/10.1007/b97636
