# Supplementary material for: Non-contact detection of pyrethroids widely used in vector control by Anopheles mosquitoes
Source: PLoS One. 2024 Jul 12;19(7):e0298512. doi: 10.1371/journal.pone.0298512 (PMC11244766; doi:10.1371/journal.pone.0298512)
Supplement: Attachment — Submitted filename: Response to Reviewers.docx [file pone.0298512.s002.docx]

**Response to Reviewers**

*May, 22^nd^, 2024*

**Manuscript Number**: PONE-D-24-03507

**Title**: Non-contact detection of pyrethroids widely used in vector control by *Anopheles* mosquitoes

*Dear academic editor and reviewer*,

Thank you for giving us the opportunity to submit a revised version of our manuscript entitled: “Non-contact detection of pyrethroids widely used in vector control by *Anopheles* mosquitoes”. We appreciate the time and effort that you and the reviewer have dedicated to providing valuable feedback on our manuscript. We are grateful to the reviewer for his insightful comments on our paper. We have been able to incorporate into this new revised version of the manuscript the suggestions provided by the reviewer. We have highlighted the changes within the manuscript.

Below is a point-by-point response to the reviewer’s comments and concerns.

**Response to Reviewer #1**

Comments of reviewer 1 do not concern our paper and we notified this to Plos one which answered us through the senior editor Mr/Mrs. Miquel Vall-llosera Camps in the following words: **“**I am writing to follow up on your email copied below. First of all I would like to sincerely apologise for the delay you have incurred with your submission. I have discussed your concerns with the Academic Editor and we consider that, for this revision round, please only address the comments by Reviewer#2. We have suggested that Reviewer#1 should not be invited to next rounds of review and the Academic Editor might decide to invite additional reviewers if required. If you have any questions or concerns that you would like to discuss further please do let me know**”**.

**Response to Reviewer #2**

- **Introduction**

*Reviewer comment 1*: In line 47 it’s wrote '' by decreasing both human-vector'' ref 2, this corresponds well to the reference which talks about mosquito nets. However, i would suggest that the author check whether it is pyrethroids that are mentioned in the report for reducing human-vector contact or mosquito nets.

**Response**: Thank you for this remark. We agree with you that the cited study is about ITNs but not directly pyrethroid insecticides and the efficacy of ITN was already mentioned in the first sentence. To avoid redundancy, we removed this sentence that was not informative.

- **Material and methods**

*Reviewer comment 2*: Line 113, the author used females aged 4-8 days and not fed blood. The author should explain in one sentence why he preferred this age group of mosquitoes for the tests.

A map of the study area where the field mosquito larvae were collected would be more explicit.

**Response**: Thank you for pointing this out. We now bring explanation for why we used mosquitoes aged from 4-8 in lines 123, 124 and 125 of the manuscript track-change version.

Concerning the study area where the field mosquito larvae were collected, we consider that the GPS coordinates that we provided are sufficient as all the larvae were collected from a single larval site location.

*Reviewer comment 3:* On line 149, the author states that the permethrin test on laboratory mosquitoes was carried out at the IRD in France? no equipment was needed to carry out this test in the laboratory in Burkina Faso? In other words, why weren't the tests carried out in a single laboratory because we found that the take-off rate of mosquitoes in the control group was lower with permethrin than with other insecticides, where it was higher. It would be important to clarify whether there is a particular explanation for this.

**Response**:

Thank you for the question. The experimental protocol was initially developed in France using laboratory colonies (i.e., Kisumu and KDR-Kisumu) and permethrin, and then moved to Burkina Faso to test it in wild mosquitoes. Once there, we took the opportunity to test other insecticides with laboratory colonies. Indeed, in our study there is a confounding effect between insecticide molecule and location mainly due to the behavioral response differences between controls (i.e., permethrin vs. deltamethrin and cypermethrin), even though the experiments were performed by the same person, environmental conditions in both laboratories were standardized, and the material used and mosquito strains were exactly the same. Despite these efforts, location-dependent conditions may still exist (atmospheric pressure, water quality, etc.). However, the statistical analyses were made taking high care of these differences between sites (see statistical analysis section “Consequently, the data associated with each insecticide were analyzed separately. Line 198) and we believe that we did not over interpret our results. In the revised version, we just added “for project related reasons” from line 175 to 176 in the track change version.

*Reviewer comment 4:* Lines 149-151 the author wrote: ''Assays involving permethrin on laboratory strains were carried out in Montpellier, France. Assays involving deltamethrin and alpha-cypermethrin on laboratory and field collected mosquitoes were carried out in Bobo Dioulasso, Burkina Faso''. However, the results showed that the field strains were only used for exposure to permethrin. If the results for the other two insecticides are not available, it would be better to correct this sentence to ensure consistency in the results.

**Response**: Thank you for this great remark. We added one sentence from line 178 to 179 in the track change version of the manuscript to ensure the consistency with the results.

*Reviewer comment 5:* All the additional figures are not at all legible, really small in size and do not properly facilitate understanding of the protocol.

**Response**: Thank you for your remark. We modified the size of the additional figures accordingly in supplementary materials track change version going from line 69 to163.

- **Result**s

*Reviewer comment 6:* Lines 201-205 have no place in the results and have already been described in the material and methods.

**Response**: We agree and we removed these lines (first paragraph of the results section) following your comment as it looks like a repetition.

*Reviewer comment 7:* Lines 272, 355 and 361 (in discussion): the author wrote ''Gambiae complex'' or ''Gambiae''; I believe the author meant to write An. gambiae complex. If so, the author should correct these sentences.

**Response**: Thank you for rising this point. After re-checking the current taxonomic literature, the consensual form of citing species complexes would be in our case: “Gambiae Complex”, both words written with a starting capital letter (see Harbach and Wilkerson, 2023, Zootaxa, 5303, Magnolia Press. <https://doi.org/10.11646/zootaxa.5303.1.1>). We have changed accordingly at lines: 25, 34, 352, 454-455 and 472 in the track change version of the manuscript.

- **Discussion**

*Reviewer comment 8:* In the discussion section, I expected the author to clarify the limitations of the control tests used given that the mosquito take-off rate was too low (less than 5%). Although it is true that the difference is significant compared to the treated bottles, other non-toxic products could activate the same reflexes in mosquitoes and it would be important to clarify this.

**Response**: In fact, we do not consider that the control results in the permethrin experiment are too low, but rather that the controls in deltamethrin/cypermethrin experiments are considerably high. We used acetone (the solvent used for insecticide dilutions) as control. Bottles containing the chemicals were left under the chemical hood until all the solvent was evaporated. Thus, in principle, only trace amounts of the solvent could still be present in the bottle. We have repeated the same protocol for other experiments (Kambou et al., unpublished data) using acetone and other solvents, and the control results are similar to those observed in the permethrin (France) experiment, and we have considered these results as spontaneous take-off activity with the given conditions. Unfortunately, we still do not have an explanation for why control tests in Burkina Faso ended up being higher than in France. In any case, we performed control tests for each insecticide experiment. This fact allows us to analyze and interpret the results of each insecticide test without major methodological problems. Issues could arise when trying to compare the results among insecticide tests, but this idea was already discarded at the beginning of the study simply because the different experiments could not be performed in parallel (i.e., at the same time), and temporal variables, as for instance: laboratory mosquito batches, date, atmospheric pressure, etc. could not be standardized.

We agree that other compounds, independently of their toxicity, could elicit the same take-off response. Yet, in this study we do not associate take-off response with compound toxicity, besides we consider that the experimental protocol is not suited to test this question. This experimental protocol is suited to assess a sensory detection of one stimulus (in this case chemical compounds) by mosquitoes if, and only if, there is a take-off response associated. In the absence of a behavioral response, therefore, we cannot determine that a sensory detection of the stimuli exists.

*Reviewer comment 9:* For clarification, was the exposure time (30 seconds) perfectly ideal? Why not shorten it by 15s or perhaps go to 1min? Could someone repeat the tests under the same conditions but slightly modify the exposure time to obtain the same result? If not, it would be more appropriate to set out the arguments in one sentence.

**Response**: Thank you for raising the fact that we forgot to mention the origin of our protocol. Our protocol was inspired from a previously developed assay, for testing detection of repellents (Afify et al., 2019, Current Biology) and that was able to discriminate differential detections according to molecules and conditions. We added the reference in the new version of the manuscript (line 159 in track change version- reference [49]). Nonetheless, we agree that recording the take-off time within these 30s exposure would have provided us with some temporal response information during this time period. To answer your questions: i) we did not test other exposure times, so we do not know if 30 s is the most optimal exposure time. ii) we hypothesize that 15s would give a slightly lower number of take-off responses as most of the take-offs were usually observed few seconds after the exposure. On the other hand, using 1 minute would give equal or slightly higher take-offs, in both insecticide tests and controls. iii) we are convinced that our experiments are repeatable and we encourage other researchers to validate these (or similar) questions using the same protocol.

**Responses to the journal requirements 1**

1. When submitting your revision, we need you to address these additional requirements.

**Response:** We carefully followed the journal template and renamed all the figures and tables in the manuscript as well as in the supplementary materials accordingly (change track versions).

2. We suggest you thoroughly copyedit your manuscript for language usage, spelling, and grammar. If you do not know anyone who can help you do this, you may wish to consider employing a professional scientific editing service.

**Response:** Our manuscript was read and corrected again by one of the co-authors, Dr. Philip Agnew research engineer at IRD who works in mosquito behavior and who is also an English native speaker. He made all the necessary in terms of language usage, spelling, and grammar formulation to make the manuscript easy to read and understandable.

3. Thank you for stating the following financial disclosure: This study was financed by the French National Research Agency – ANR ([https://anr.fr](https://anr.fr/)), project INDEed (ANR-21-CE35-0021-01) granted to DC. The “Institut de Recherche pour le Développement” through the program ARTS provided funding for SSK doctoral studies.

**Response:** We included the following statement: "The funders had no role in study design, data collection and analysis, decision to publish, or preparation of the manuscript." in the cover letter new version and in the new version of the manuscript under the “Funding” section (from line 495 to 496 in the track change version).

4. Thank you for uploading your study's underlying data set. Unfortunately, the repository you have noted in your Data Availability statement does not qualify as an acceptable data repository according to PLOS's standards.

**Response:** Please note the repository is listed with the 'FAIRsharing and 're3data' organisations (see links below) and is powered by 'The Dataverse Project' software developed at Harvard University.

- FAIRsharing.org - entry 'DataSuds': <https://fairsharing.org/FAIRsharing.7c255a>
- re3data.org - entry 'Datasuds': <https://www.re3data.org/search?query=DataSuds>

It is maintained by the 'Institut de Recherche pour le Developpement' (IRD), a national research institute funded by the French government.

Data in the repository have a 'DOI' and are freely available to the public with 'CC BY' licences. Furthermore, several items already in the repository are associated with papers published in 'PLOS One' or other 'PLOS Journals' (see link below).

DataSuds respository, search term 'PLOS' :

<https://dataverse.ird.fr/dataverse/root?q=PLOS>

Consequently, we think the repository meets the criteria suggested by PLOS and have deposited our data there.

NOTE: The following is a confidential temporary link to the dataset deposited in 'Datasuds' that Editors and Reviewers can use to see the files:

<https://dataverse.ird.fr/privateurl.xhtml?token=0e49d898-0890-49fa-a207-a25faa33beaf>

A permanent 'DOI' has been already attributed to the dataset and it will remain unchanged: DOI: <https://doi.org/10.23708/IMJ1WG> . This 'DOI' is given in the data availability statement in the manuscript. The DOI can only be activated once the manuscript is accepted for publication, otherwise any changes proposed by either the editor or the reviewer on this datafile would not be possible after its validation.

We added a new section entitled Data availability in the revised manuscript (from line 498 to 501 in the track change version).

5. Please include captions for your Supporting Information files at the end of your manuscript, and update any in-text citations to match accordingly.

**Response:** We have taken this remark into account, as you can see from lines 452 to 453 in the track change version of the manuscript.

6. Please review your reference list to ensure that it is complete and correct. If you have cited papers that have been retracted, please include the rationale for doing so in the manuscript text, or remove these references and replace them with relevant current references. Any changes to the reference list should be mentioned in the rebuttal letter that accompanies your revised manuscript. If you need to cite a retracted article, indicate the article’s retracted status in the References list and also include a citation and full reference for the retraction notice.

**Response:** We reviewed our reference list to ensure that it is complete and correct and we added 3 new references (references [2], [45] and [49]) highlighted in yellow the new version of the manuscript track change version in order into account one of the reviewer 2 comments.

**Responses to the journal requirements 2**

1. Please remove your figures/ from within your manuscript file, leaving only the individual TIFF/EPS image files.  These will be automatically included in the reviewer’s PDF

**Response**: We removed all the figures accordingly in the manuscript new version and leave the image files in TIFF formats

2. We note you have provided the following data link:

<https://dataverse.ird.fr/privateurl.xhtml?token=0e49d898-0890-49fa-a207-a25faa33beaf>

We also note this data set is unpublished and private. Before we can proceed, please unlock this data set so that it is public and has a permanent DOI.

**Response**: Please note we can and will provide a permanent DOI to the files we have deposited at the DataSuds **repository once our article has been accepted**. Until such time, we have provided an unpublished and private link that Editors and reviewers can use to access the files in the repository. This follows the submission guidelines provided by PLOS :-

The unpublished nature of the link means we can modify the contents of the files in response to any points raised by the reviewers, e.g., if we need to change an analysis and consequently the corresponding file in the repository containing the R-script.

Our 'Data availability statement' now reads

The data [and related documentations] that support the findings of this study are openly available in DataSuds repository (IRD, France) at [*https://dataverse.ird.fr/privateurl.xhtml?token=0e49d898-0890-49fa-a207-a25faa33beaf*](https://dataverse.ird.fr/privateurl.xhtml?token=0e49d898-0890-49fa-a207-a25faa33beaf). Data and documentations reuse is granted under CC-BY license. R script is granted under GPLV3 license.

We look forward to hearing from you in due time regarding our submission and to respond to any further questions and comments you may have.

Sincerely,

Sassan Simplice KAMBOU
